# Assessing the Acceptability of a Preschool-Based Multi-Component Physical Activity Intervention Entitled “I’m an Active Hero” (IAAH): Process Evaluation of a Feasibility Trial

**DOI:** 10.3390/healthcare12141398

**Published:** 2024-07-12

**Authors:** Mosfer A. Al-walah, Shayek S. Alotaibi, Adel A. Alhusaini, Meteb M. Alotiabi, Michael Donnelly, Neil Heron

**Affiliations:** 1 Centre for Public Health, School of Medicine, Dentistry and Biomedical Sciences, Queen’s University Belfast, Belfast BT12 6BA, UK; 2 Department of Physical Therapy, College of Applied Medical Sciences, Taif University, Taif 21974, Saudi Arabia; 3 Department of Medical Rehabilitation and Physiotherapy, Children Hospital, Ministry of Health, Taif 26524, Saudi Arabia; 4 Department of Rehabilitation Sciences, College of Applied Medical Sciences, King Saud University, Riyadh 11433, Saudi Arabia; 5 School of Medicine, Keele University, Keele ST5 5BG, UK

**Keywords:** childhood obesity, physical activity, intervention, preschool, children, Saudi Arabia

## Abstract

Background: Interventions within preschool settings have gained prominence due to the need to increase physical activity (PA) in early childhood. We first developed a 10-week preschool-based behaviour change intervention, guided by the UK Medical Research Council’s framework for complex interventions. We then conducted a cluster feasibility randomised controlled trial (RCT) among young children. Aim: This process evaluation was embedded within the cluster feasibility RCT and aimed to assess the acceptability of the 10-week IAAH intervention among both preschool staff and parents. Methods: The study utilised a mixed method, involving post-intervention questionnaires completed by preschool staff (n = 4) and children’s parents/caregivers (n = 9) and focus groups with preschool staff (n = 3) and parents/caregivers (n = 7). Quantitative data were analysed using SPSS to calculate acceptability scores, while qualitative data underwent thematic analysis using NVivo 12. Results: The intervention was well-received, with preschool staff reporting a 94.5% acceptability rate (mean score of 10.4 out of 11) and parents/caregivers indicating an 86% acceptance rate (mean score of 5.2 out of 6). Thematic analysis of focus group discussions revealed facilitators to intervention delivery, such as user-friendly materials and alignment with preschool curricula, and identified barriers, including time constraints, spatial limitations, and policy conflicts. Parental engagement was hindered by time restrictions, although the intervention materials were praised for their clarity and visual appeal. Conclusions: The findings suggest that the IAAH programme was acceptable to both preschool staff and parents. However, the identified barriers to intervention delivery and engagement should be addressed in the planning of a future cluster RCT to evaluate the efficacy of the intervention.

## 1. Introduction

Physical activity is integral to the holistic well-being of young children, encompassing their physical, mental, and psychosocial health [1]. The benefits of engaging in physical activity during childhood extend across various short-term and long-term health dimensions [2]. Global guidelines have underscored the importance of preschoolers engaging in a minimum of 180 min of moderate-to-vigorous physical activity (MVPA) daily, while minimising sedentary time, for optimal health outcomes [3,4,5,6]. Unfortunately, empirical data have revealed that less than half of preschoolers adhere to these recommended guidelines, necessitating the implementation of strategies to boost physical activity and mitigate early childhood obesity [7,8,9].

Aligned with public health directives advocating for early physical activity interventions [10,11], the early years of childhood offer a critical window for such intervention [12]. Kindergartens, in particular, present an opportune environment for promoting physical activity among children aged 3–5 years given the substantial time they spend in these settings [13,14].

Despite the emergence of intervention programmes targeting physical activity among preschool children, outcomes have varied [15,16,17]. Notably, these interventions have primarily been implemented in developed countries, with a scarcity of such initiatives in developing nations like Saudi Arabia [17]. While some studies in Saudi Arabia have focused on increasing physical activity among primary-school and adolescent students [18], there remains a dearth of evidence regarding the feasibility and effectiveness of theoretical interventions designed for young children. Our study addresses this gap by introducing the “I’m an Active Hero” (IAAH) preschool-based multi-component physical activity intervention in Taif City, Saudi Arabia [19], and conducting a feasibility cluster randomised controlled trial (cRCT) that included an assessment of acceptability.

Traditionally, evaluations of complex interventions like “I’m an Active Hero” (IAAH) have emphasised the measurement of effectiveness per se, overlooking the crucial aspect of intervention or programme acceptability [20,21]. This oversight persists despite recommendations from the UK Medical Research Council (MRC) to evaluate feasibility and acceptability during the development and assessment of complex interventions [22]. Process evaluations play a vital role in understanding the delivery and execution of intervention components, offering insights into positive or negative outcomes [23]. However, there is a notable paucity of evidence regarding process evaluation in previous physical activity interventions for preschool-aged children [24].

Therefore, the primary aim of our study was to assess the acceptability of the IAAH intervention and various outcome measures within a preschool setting in Taif City, Saudi Arabia. Our evaluation of acceptability encompassed diverse facets, including experiences of delivering the intervention as reported by preschool educators, the reception and execution of the intervention as reported by parents, and specific issues related to outcome measurements during the feasibility cRCT.

## 2. Materials and Methods

### 2.1. Study Design, Setting, Period, and Reporting

A mixed-methods approach was employed in this study, utilising questionnaires and semi-structured focus group discussions (FGDs) in the post-intervention phase. As part of a feasibility cluster randomised controlled trial (cRCT) conducted in two preschools in Taif City, Saudi Arabia, the “I’m an Active Hero” (IAAH) intervention was implemented in one preschool. Children aged 3–5 years were recruited from preschool classrooms [19]. In this mixed-methods study, data were collected from various groups of preschool practitioners and parents/caregivers at the end of the intervention (from April 2023 to May 2023, by prior appointment). The process evaluation followed the MRC framework [25]. The qualitative aspect of the acceptability study adhered to the Consolidated Criteria for Reporting Qualitative Research (COREQ-32) guidelines [26] (see Supplementary File S1). 

### 2.2. The IAAH Intervention

Detailed information about the specific components, content, and activities of the IAAH programme can be found in a separate publication designed to facilitate a comprehensive understanding of the intervention’s elements [19]. The IAAH programme, a comprehensive 10-week initiative implemented in preschools, aims at promoting physical activity and reducing sedentary behaviours among children aged 3–5 years with family involvement. This programme was developed in alignment with the Medical Research Council’s (MRC) Framework for the Development and Evaluation of Complex Interventions [22], guided by social cognitive theory and the social ecological model as the theoretical frameworks. The development process was systematic, involving stages such as conducting an in-depth review to identify effective behavioural change techniques and integrating insights from key stakeholders and experts in the field. Supported by the lead researcher, preschool teachers participated in two preparatory sessions to ensure the initiative’s consistent and effective execution. The face-to-face delivery method, selected for its proven efficacy in prior studies [27,28], supported the programme’s focus on crucial aspects of energy balance: increasing physical activity and reducing sedentary time. Intervention components, designed for use in both preschool and home settings, included staff training, environmental adjustments, structured physical activity sessions, and classroom movement breaks. For families, the materials included newsletters, tip cards, and posters designed to enhance physical activity behaviours in preschool-aged children. In addition to these educational materials, families were provided with various ideas about physical activity, suggestions for daily family games, and interactive activities requiring active participation and goal setting from both parents and children. 

### 2.3. Outcome Measures

The outcome measures for the trial are provided elsewhere [19]; briefly, they included the intervention’s feasibility, fidelity, recruitment, and attrition rates; compliance with procedures; and objective measurements of children’s physical activity and sedentary time using the ActiGraph GT3X accelerometer, alongside assessments of children’s height and weight to calculate the standardised Body Mass Index (zBMI).

### 2.4. Study Participants and Recruitment

Teachers and parents who participated in the cluster feasibility RCT and consented to take part in this post-intervention section of the study were eligible for focus group participation. Invitations were extended through preschool head teachers, who distributed information sheets and consent forms. For parents, a purposive sampling method was employed, and the practitioners facilitated the dissemination of information sheets and consent forms prior to the focus group discussions.

### 2.5. Focus Group Procedures and Transcription

Prior to starting the focus groups, their purpose and procedures were explained to the participants (including both teachers and parents). They were informed that their participation was voluntary, that they could stop at any time, and that they would be given the opportunity to ask questions. The focus group facilitator (MA) was a trained researcher (male) who had no prior relationship with the participants. The facilitator used open-ended questions and reminded participants that there were no right or wrong answers in order to encourage open dialogue. The researcher took notes for coding and theme generation. With consent, a Dictaphone digital recorder was used to audio-record all focus groups. These recordings were transcribed verbatim for further analysis while ensuring participant anonymity. To further ensure accuracy and completeness, two researchers (MA and NH) thoroughly reviewed the transcripts against the original audio recordings.

#### 2.5.1. Intervention Acceptability—Preschool Component

A focus group with teachers was conducted in the staff room of the intervention preschool. Four preschool staff who facilitated the IAAH intervention (3 teachers and 1 assistant teacher) were invited to participate in the focus groups. Three teachers consented and participated, while one assistant teacher did not participate due to other commitments. The focus groups, lasting approximately 45 min, followed a semi-structured guide (see Supplementary File S2) with questions examining barriers and facilitators to implementing the intervention and RCT. Discussions also explored areas for enhancement in future implementation. Additionally, participating teachers completed post-intervention forms with Likert scale and open-ended questions to gauge perspectives on the intervention and identify areas for improvement.

#### 2.5.2. Intervention Acceptability—Home Component

Parent focus groups were conducted online via Zoom—a method chosen for its convenience and accessibility, particularly considering evidence suggesting that virtual focus groups can effectively facilitate participation from diverse geographic locations while reducing logistical burdens such as travel time and costs. Of the 20 parents originally invited to participate, 11 (55%) consented to take part. Ultimately, 7 individuals (3 mothers and 4 fathers) attended the sessions. However, despite rigorous organisation, reminder phone calls, and incentives such as shopping vouchers, none of the remaining parents turned up to the scheduled session. Several factors were cited that prevented participation, including time constraints, privacy concerns, and lack of interest. The focus group, following a topic guide (see Supplementary File S3), explored parents’ views and experiences of the IAAH home component. It also examined barriers and facilitators to participation. Sessions lasted 45–55 min and were audio recorded and transcribed verbatim. Parents/caregivers received a GBP 25 shopping voucher as compensation for participating. Additionally, teachers distributed post-intervention feedback questionnaires to all intervention preschool parents.

### 2.6. Ethics

Ethical approval was obtained from the Saudi Arabian Ministry of Health’s Research and Studies Department (IRB registration number with King Abdulaziz City for Science and Technology (KACST), KSA: HAP-02-T-067).

### 2.7. Data Analysis

To assess acceptability in this study, we coded responses to post-intervention questionnaires similarly to the approaches used by previous studies [27,28,29]. For dichotomous items, a positive response (yes) received a code of 1, while a negative response (no) was coded as 0. For Likert scale items, a response of 4 (agree/often) or 5 (strongly agree/always) was coded as 1, while all other responses (1–3) were coded as 0. Total possible acceptability scores of 11 and 6 were available for practitioners and parents, respectively. We calculated proportions to determine the percentage acceptability score for each sample, which was based on the model of Saunders et al. (2005) [30,31]. Since there are currently no established acceptability quantification guidelines, we used Durlak and DuPre’s (2008) [23] fidelity scoring recommendations, categorising ≥60% as high acceptability.

All focus group sessions were transcribed word for word and rendered anonymous prior to analysis. We analysed the focus group data utilising the six phases of thematic analysis outlined by Braun and Clarke, employing a realist method [32]. Notably, this analysis method is recursive; each phase might necessitate a return to prior phases, prompted by emerging themes, insights, or data [33]. In phase 1 (familiarising themselves with the data), the researchers (MA and NH) familiarised themselves with the data by examining and re-examining the transcripts and audiotapes. During this phase, initial ideas for coding were discussed (MA and NH). In phase 2 (generating initial codes), transcripts were systematically and independently coded by the researchers (MA and NH), with initial codes generated to structure the data around potential topics of interest [33]. One focus group discussion was coded by both researchers (MA and NH). Following this, the coders discussed and defined a coding framework for the remaining dataset, noting any patterns or discrepancies among the codes [32]. All coding disagreements were resolved through discussion between the coders. In phase 3 (theme identification), the coded data from all focus groups were collectively reviewed (MA and NH) to develop themes. Themes were inductively [34] and iteratively created by comparing, analysing, combining, and mapping the codes [33]. An initial delineation of possible themes and subthemes was composed and discussed (MA, NH, and MD). Phase 4 (reviewing themes) involved assessing the potential themes against the transcripts to confirm their validity across the entire dataset. Themes were then refined further (MA and NH). In phase five (defining and naming themes), each theme was elaborated upon using the transcripts, whereupon themes were named and defined (MA and NH). The final phase, phase 6 (producing the report), involved drafting the report and selecting illustrative quotes to underscore the themes (MA, NH, and MD). The report we composed reflects the continuous iterative interpretation and analytical process of thematic analysis [35].

Transcripts from the focus group with teachers were analysed and coded first, followed by transcripts of the focus group with parents. Similar codes from both the teachers’ and parents’ focus groups were then collated together, leading to the emergence of themes and subthemes within each category. In addition, specific codes relating only to teachers or parents were grouped based on similarity of meaning (for example, codes related to the activity diary for parents or the physical activity booklet for teachers). The identification of specific teacher and parent themes and subthemes was based on the similarity in meaning of these unique codes. Additional data from practitioner logbooks and parent questionnaires were also incorporated during the coding process. NVivo version 12 software was employed to enable data storage and organisation for analysis and method triangulation, collectively examining the quantitative questionnaires and qualitative focus groups. 

Reaching data saturation is essential in qualitative research. As Saunders et al. (2017) explained, there is no single point at which saturation is achieved across all studies [36]. To determine whether saturation was reached in this study, emerging themes were systematically identified during data analysis. Saturation was demonstrated when additional data collection and reviewing revealed no new substantive themes [37]. This iterative approach balances gathering sufficiently rich data with pragmatic constraints. In their systematic review, Hennink and Kaiser (2022) emphasised the primacy of data quality over participant quantity in qualitative research, advocating sample sizes that ensure detailed and accurate representation of the subject matter. They noted that saturation typically occurs within 4–8 focus groups, although variations can range from 2 to 40 groups [32], as it is influenced by the homogeneity of the population and the specificity of the research goals. Studies targeting more uniform groups with narrower questions tend to reach saturation with fewer groups, while those exploring diverse populations and broader questions require larger samples [32]. Accordingly, the sample sizes in our study, such as those determined for focus groups, lay within the aforementioned range and utilised homogeneous samples with defined aims and proved to be efficient, especially given that the focus was on data depth and richness rather than sheer quantity. However, determining the appropriate sample size in qualitative research involves considering the study’s objectives, the phenomenon’s complexity, the instrument’s structure, and the sampling strategy. Factors such as sample stratification, researcher expertise, saturation goals, and the desired saturation level also significantly influence this decision [33].

## 3. Results

The results of this study yielded two types of data: quantitative data resulting from the post-intervention questionnaire and qualitative data from the focus groups. These are reported further below in the Acceptability Questionnaires and Main Themes subsections, respectively. 

### 3.1. Acceptability Questionnaires

Following the intervention, post-intervention questionnaires were successfully completed by three out of the four preschool staff members, all of whom also participated in the subsequent focus group discussions (Table 1). The questionnaires were also disseminated to the parents/guardians of the 20 eligible participants, resulting in 14 completed questionnaires. After excluding five incomplete responses, a total of nine questionnaires were deemed suitable for analysis. Invitations to join the focus group discussions were extended to all 20 parents. Initially, 11 parents expressed interest, and ultimately 7 parents (comprising 3 mothers and 4 fathers) agreed to take part in these conversations (Table 2).

### 3.2. Preschool Acceptability and Main Themes

The total acceptability score for the post-intervention practitioner questionnaire responses was 94.5% (mean score: 10.4/11), indicating that the acceptability of the intervention was high. 

As the interpretation and thematic analysis of the focus groups with teachers and parents revealed remarkable overlaps in the resulting themes (Table 3), the results from staff and parents were presented and discussed together. Overall, there were three main themes: the acceptability of the trial procedures, the outcomes of the intervention, and perceived challenges and the potential for sustainability. Each main theme is summarised below and is illustrated using examples of relevant quotes from the focus groups.

### 3.3. The Need for Interventions to Increase Physical Activity in Saudi Preschools

#### Increasing Opportunities

There was widespread agreement among practitioners and parents that initiatives like the IAAH are essential for Saudi preschools. They pointed to alarming rates of childhood obesity, a rising dependence on screen devices, and limited access to opportunities to engage in healthy behaviours as compelling reasons for implementing the programme:

“In fact, this is one of the first programmes that we have come across, and I think it is useful, and I believe that there is a need for such good and useful programmes, especially for children who live in apartments with limited space to play outdoors. Nowadays, parents are reluctant to let their children roam freely due to safety concerns, which leads to decreased physical activity. Many children are glued to their electronic devices instead of engaging in active play. Hence, I saw it as a good and positive option for children.”

(Father 3)

### 3.4. Acceptability and Feasibility

#### 3.4.1. Practitioner Perceptions of Intervention Content and Materials

Overall, practitioners viewed the intervention as both acceptable and manageable to implement. They identified various factors that either hindered or facilitated its implementation. Notably, they found the classroom materials and activity guides to be valuable resources, simplifying the delivery of physical activity sessions by minimising the need for extensive pre-planning:

“Regarding the programme, it was good. I’ve spoken about this before and praised it. The programme was very good. Yes, there were some minor obstacles, but overall, the programme was excellent and new for our context.”

(Teacher 2)

All participants agreed that the classroom materials were visually captivating; contained clear, concise instructions; and used appropriate language—all of which contributed to the successful implementation of the programme. Practitioners also expressed that the classroom activity guides provided flexibility in delivery, allowing physical activity sessions to be easily customised to fit the unique context of each preschool:

“The programme guide was very useful and clear. The content was straightforward, and the steps were sequential and detailed. It also included illustrative examples. It presented realistic and illustrative examples showing how to apply the programme in classrooms, supported by pictures. Also, I shouldn’t forget the attractive design of the booklet. It was appealing, colourful, and clear. It used illustrative images and coordinated colours that reflected the idea of the programme.”

(Teacher 3)

#### 3.4.2. Parent and Child Perceptions

Several practitioners mentioned that they had received feedback from parents about the changes made in the preschool and the activities designed for parent–child interaction as part of the intervention. However, some practitioners noted that they had not received any feedback from parents. Generally, the feedback from parents was positive, particularly regarding the benefits of the intervention’s home-based activities:

“For the parents, I noticed a positive interaction from them with the parts of the programme at home. They participated with their children in exercises and activities and expressed their admiration for the ideas and suggestions provided to promote physical activity at home. Although some of them said that family obligations, lack of time, and the social way of life in general affected the implementation of the programme, as it should, they were enthusiastic and tried to implement it as much as they could.”

(Teacher 1)

“As for the parents, they had positive reactions to the programme. Most of them showed great interest in the household items that were distributed to them. They benefited from tips and ideas to encourage physical activity at home, and some of them said that their children’s lifestyle improved and increased in terms of activity, movement, and enthusiasm.”

(Teacher 3)

Although some practitioners at the preschool reported not receiving direct feedback from parents, they learned from children that the home-based materials were being used by the children and their parents:

“Even if there are difficulties, that is, if there is a shortage of classes and a shortage of staff, it is a problem in time, but there is a benefit to it. The children accepted the programme. The children became happy. The children became leaders of another group.”

(Teacher 1)

The children embraced the programme, demonstrating happiness, leadership, and a desire to repeat favourite activities, indicating how they enjoyed the preschool-based aspects of the intervention, as reported by practitioners based on the children’s enthusiastic participation and requests.

#### 3.4.3. Training of Practitioners

The training session conducted prior to the intervention was perceived positively by participants. They reported that it was adequately informative and provided them with the necessary skills and knowledge to effectively carry out the intervention within the preschool setting:

“As a kindergarten teacher, I see that the training we received was wonderful, beautiful, and beneficial.”

(Teacher 2)

“We benefit from the training courses, and, as my colleague said, not only did the staff benefit in general, but we also saw the children benefit from the programme.”

(Teacher 3)

#### 3.4.4. Adaptation to Environmental Changes in Preschool Intervention Classrooms

The acceptance of environmental modifications within preschool classrooms involved in the intervention exhibited variability, with participants encountering several challenges. Facilitators identified spatial constraints and discrepancies with existing preschool policies as significant hurdles. Nevertheless, participants demonstrated adaptability, employing a variety of strategies that were specifically designed to align with their unique skills and methods:

“I tried to choose a wider classroom so I could arrange it in a meaningful way and allocate a part of the classroom as a space for better movement and activity.”

(Teacher 1)

“We adjusted some activities suitable for the classroom, such as imitating animals and other simple games that align with the available space. We also attempted to create a more spacious environment by rearranging the classroom, allowing children to move around easily.”

(Teacher 2)

#### 3.4.5. Mode of Delivery

Practitioners’ approaches to delivering the intervention varied, being influenced by their individual capabilities and the need to coordinate with other schedules, classes, and administrative duties in different preschool environments. All practitioners implemented the programme both indoors and outdoors, contingent upon favourable weather conditions. Furthermore, rather than conducting sessions continuously from start to finish, some practitioners preferred to segment the sessions, distributing them at different intervals throughout the day:

“Of course, commenting on what the teachers said, there was indeed difficulty in the beginning when implementing any new idea or programme. For me, I allocated 5 min from some lessons to implement the programme inside the classroom. Sometimes I give the children the full session in the kindergarten’s inner courtyard so they can have more fun.”

(Teacher 3)

Practitioners consistently observed that children required minimal instruction to engage in the sessions—a factor they regarded as beneficial. This ease of participation aligns with the intervention’s emphasis on child-led activities, which is a core component of the programme’s design. Such an approach not only fosters autonomy but also enhances the effectiveness of the intervention by actively involving the children in the learning process:

“We didn’t require staff supervision; the children were eager to engage on their own. They often added their own unique touches to the activities, which was wonderful to observe.”

(Teacher 1)

#### 3.4.6. Implementation of Physical Activity Sessions by Practitioners

Practitioners expressed a high level of enjoyment in delivering the physical activity sessions, which was evident in their robust implementation efforts:

“Each person has their own personal experience and circumstances. Honestly, it was the first time I encountered such a programme, despite having over ten years of experience in kindergarten. I was enthusiastic about reading and exploring all parts of the programme.”

(Teacher 2)

“There is enthusiasm for implementing the programme, but our buildings need proper preparation for such programmes.”

(Teacher 3)

### 3.5. Trial Procedures

#### Acceptance of Accelerometers

The pilot procedures implemented to evaluate the feasibility of the IAAH study were generally well received by practitioners, who found the duration of data collection to be reasonable. However, of universal interest among participants was the acceptability of the ActiGraph GT3X accelerometers. Although the children were enthusiastic about using these devices, practitioners considered them to be overly intrusive. Some parents, in particular, expressed reservations because the devices had to be worn for a long period of time and were uncomfortable for their children:

“The children accepted all the ideas and parts of the programme, and there was some reluctance to wear a belt or device before the programme, so there was encouragement.”

(Teacher 2)

“Some of the children did not welcome or were generally bothered by placing the device to measure the child’s movement or activity.”

(Teacher 1)

Practitioners unanimously recommended considering alternative devices that would be less disruptive and more convenient for parents. Options such as accelerometers worn on the wrist or ankle have been suggested as more suitable alternatives for the target population. In addition, the possibility of incorporating mobile phone technologies for short-term monitoring has also been suggested, encouraging a shift towards more user-friendly methods of data collection in future applications of the study:

“To solve the problem of non-compliance with the measuring device, I expect that if the period were shorter or if it were possible to use technology, for example, there would be an application on the mobile phone that would be more welcome.”

(Teacher 2)

### 3.6. Home Acceptability

Parental feedback collected through post-intervention questionnaires indicated a high level of acceptability, with a total acceptance rate of 86% (mean score of 5.2 out of 6). Table 4 provides a detailed breakdown of parental responses to specific acceptability criteria within the questionnaire. Responses concerning the acceptability of materials and activities (Items 1, 4, 5, and 6) showed higher ratings compared to those concerned with the intervention’s perceived impact on health behaviours (Items 2–3). The findings from the focus groups are also detailed below.

### 3.7. Acceptability of Intervention Materials

#### 3.7.1. Parental Perceptions of Materials and Activities

Parents expressed satisfaction with the visual design of the materials. They found the duration of the activity packs to be adequate and noted that the language was simple to comprehend, with instructions that were straightforward and easy to execute:

“In fact, the programme is already easy by nature. There are no real difficulties, and all the materials are clear and straightforward. Personally, I find it very easy, and I don’t see anything that could make the programme easier. In fact, I believe it is already very clear and beautiful. It’s easily accessible, not costly, and doesn’t require effort from the child or the parent.”

(Mother 2)

#### 3.7.2. Child Perceptions

Parents unanimously reported that their children were enthusiastic about participating in the activities, and they responded positively to the posters and accompanying materials. Furthermore, allowing children to lead some of the activities enhanced their excitement and boosted their self-confidence, as observed by the parents:

“Yes, there is no doubt at all that I noticed that the children greatly enjoyed the “I Am the Active Hero” programme. The activities and exercises presented in the programme were full of fun and challenges that caught their attention and encouraged them to participate actively.”

(Father 4)

### 3.8. Trial Procedures

#### Acceptability of Accelerometers

Out of the 52 parents participating in the cRCT, 29 reported that their children used the accelerometer throughout the study. These parents provided valuable feedback on the device’s acceptability. While children were generally happy to wear the device, the majority of parents believed that a wrist-worn device would be more favourably received and might encourage greater parental consent for participation. Notably, one parent mentioned that their child experienced a mild rash from wearing the ActiGraph GT3X:

“I can add to what my colleague mentioned. There was some difficulty with the activity tracking device, which is the physical activity belt. The child felt restricted when wearing it throughout the day. In most cases, he resisted wearing the device because he had to wear it for four consecutive days. If it was on the hand or on the mobile phone, it might increase adherence.”

(Mother 1)

### 3.9. Outcomes and Impacts of the Intervention

Both the parents and teachers emphasised the positive outcomes of the intervention, including changes to sedentary behaviour and enhanced focus, increased enjoyment for children, and impacts on awareness and lifestyle:

“[My son] was glued to electronic devices. After this programme, he changed, increased his physical activity, became enthusiastic, and started challenging himself. He began to enjoy doing the exercises on his own without needing any assistance.”

(Mother 3)

There was also special reference to perceptions of children’s leadership, confidence, and child-led activities, along with enhanced communication and family collaboration:

“My son has shown significant and noticeable changes after the programme. He used to be addicted to electronic devices, and his physical activity was low. But now, he is very active and loves physical activities. He has embraced the idea of leadership and applies it in various aspects of his life…The programme has brought about a radical and positive transformation in him.”

(Mother 3)

### 3.10. Perceived Challenges and Preliminary Indicators of Sustainability

#### 3.10.1. Barriers to Delivery

The intervention was straightforward to implement due to accessible resources and adaptable software. However, specific challenges arose in the preschool environments, primarily logistical constraints such as limited time and space. Additionally, inadequate training for practitioners and insufficient knowledge of the intervention’s details occasionally hindered its effectiveness. These issues underscore the need to tailor approaches to the unique preschool context to enhance the intervention’s success:

“The size of the classrooms, I mean the narrowness of the classrooms, is the most important thing, and the large number of students. Also, in the kindergarten, there are no dedicated buildings. The buildings are somewhat unprepared. Also, my time as a teacher is busy most of the time.”

(Teacher 2)

“Lack flexibility in the schedules; the children have fixed lessons, and the teachers are committed to a fixed schedule. Likewise, lack of awareness of the importance of such activities may be one of the barriers.”

(Teacher 1)

#### 3.10.2. Teachers’ Prospective Sustainability of IAAH

Overall, educators indicated that the implementation of the IAAH programme did not interfere with the standard curriculum. Both teachers and parents recognised the value of these interventions and advocated for their ongoing inclusion in educational settings. They emphasised the importance of equipping classrooms with the necessary resources and materials to sustain the programme beyond the initial intervention phase. Feedback from participants at both programme sites highlighted several promising elements of the IAAH initiative:

“I am in favour of the continuation of such programmes, if time and space are available, and they are added in a way that we can say more officially, until they are added in the timetables and a teacher devotes herself to them, all difficulties will be overcome.”

(Teacher 1)

“Yes, I see the continuity of this programme, as my colleagues said, because of the many benefits that we obtained during the implementation of this programme.”

(Teacher 2)

#### 3.10.3. Parents’ Prospective Sustainability of IAAH 

Several parents emphasised the importance of sustaining the IAAH programme into the future. They recommended enhanced collaboration among key stakeholders, including state institutions such as the Ministry of Health and Education, along with increased community involvement. This cooperative approach was seen as crucial for the long-term success and impact of the programme:

“From my point of view, I think that the continuation of the Active Hero programme is very, very, very important, in the future, and useful in terms of physical activity of the child, and in terms of long-lived awareness of parents and children. In terms of how it continues to be taught, I prefer it’s taught in kindergarten and elementary schools. And this way it continues and is developed from time to time. This is my opinion.”

(Mother 2)

“I see if there is no cooperation between kindergarten and home, the programme will be cut (laughter). But the truth is that it is the default that it existed only to continue. Really, I mean, it is basically now solving a big problem. I mean, today, there are many problems. Children don’t move. It now enhances the chance that the child is moving. On the contrary, I suggest that it continue.”

(Father 3)

### 3.11. Suggestions for Improvement

Along with sharing their efforts to overcome the barriers that they faced, participants made some suggestions to improve the programme and increase its potential for sustainability. These suggestions included adjusting the activities in the programme to fit available spaces, coordinating with teachers of other sections (mainly primary schools) to share their playing areas, dedicating a fixed time for the activities, and partnership with the community to utilise spaces available outside of schools and to raise public awareness. One of the considerations highlighted by participants was that the number of children who would join, should the programme continue, would increase, which would only augment the need for adequate physical space to perform the activities:

“… preparing a suitable space and comprehensive awareness for the child, the teacher, and the parents. This is what I see as a possible addition in the coming years.”

(Teacher 1)

“I could say that the importance of widespread community participation in programmes, and the involvement of various sectors, including health, government, and both business and intellectual communities, it suggests a collective effort to support and benefit from these initiatives. For example, awareness campaigns, meetings, flyers, invitations, and WhatsApp messages. Feedback and experiences from current implementations will guide future participation and programme adjustments to be better.”

(Father 2)

“Of course, I’d like to add to what my colleague said about the programme being easy. From my point of view, it would be even easier if social media were used more. Instead of using pamphlets and papers, using various social media platforms would be more convenient.”

(Father 4)

“From my point of view, I think that the continuation of the Active Hero programme is very, very important in the future and useful in terms of the physical activity of the child. I prefer that it’s taught in kindergarten and elementary schools. And this way, it continues and is developed from time to time. This is my opinion.”

(Mother 1)

## 4. Discussion

This study delved into the acceptability of the “I’m an Active Hero” (IAAH) intervention aimed at promoting physical activity in both preschool environments and at home. The collection of qualitative and quantitative data enabled an exploration of crucial facets of intervention acceptability, thereby providing valuable insights for the ongoing refinement and enhancement of the programme within Saudi preschools.

The post-intervention questionnaire results from parents and practitioners revealed high acceptability, with 94.5% endorsing the programme. Questionnaire responses and qualitative feedback highlighted several critical factors contributing to the successful implementation of the intervention. Among these were the use of easily understandable and detailed classroom manuals, the integration of the programme’s objectives with existing preschool health and well-being curricula, and the strategic avoidance of imposing extra duties or excessive paperwork on staff. This approach took on board the lessons from other studies in which the burden of paperwork and increased workload were cited as significant obstacles to intervention acceptability [34,35]. The favourable response in this study can be attributed to the thorough involvement of practitioners in the development phase of the intervention [38]—a strategy that has been underscored in related research as being crucial for enhancing the acceptability and effectiveness of educational interventions [39]. This inclusive and collaborative approach in designing and implementing the intervention not only aligned with educators’ needs but also ensured its practical feasibility and relevance in the preschool setting.

Despite the overall positive feedback, practitioners in the preschool setting identified challenges in implementing certain aspects of the intervention, particularly the modification of the classroom environment aimed at reducing sitting time and promoting more active play. This difficulty was compounded by a noticeable disparity between the implementation levels of movement breaks and structured physical activity sessions. One insightful study [34] on a teacher-led preschool physical activity programme shed light on a potential explanation of this discrepancy. It revealed that teachers erroneously believed that rearranging the classroom was necessary for each movement break, leading to infrequent implementation due to perceived disruption. Additionally, teachers expressed a need for more training specifically focused on incorporating movement breaks seamlessly into the preschool routine. Our study echoed this sentiment, with practitioners indicating that their confidence in delivering classroom physical activity improved following training and the provision of activity guides. Consequently, it suggests that future iterations of the intervention could benefit from additional training strategies aimed at seamlessly integrating movement breaks into the preschool routine without disrupting other activities. This aligns with the need expressed by practitioners for ongoing support in this specific aspect of intervention implementation. 

The acceptability of the home-based aspect of the intervention was only marginally lower (86%) than the preschool element (94.5%), as indicated by responses from parent-completed questionnaires. Additionally, qualitative data indicated that a significant obstacle was the limited time available for parents to participate in the activities—an issue frequently encountered in studies focusing on family-based obesity prevention [40,41]. This limitation may have compromised parents’ engagement in the intervention and influenced their views on its effectiveness in altering health behaviours. Despite the identified obstacles, parents positively evaluated the intervention’s materials and activities, particularly highlighting their children’s enjoyment, the effectiveness of child-led activities and poster incentives, and the clarity of the provided instructions. These responses indicate that the main issues with acceptability may relate more to the mode of delivery rather than the content itself. In contrast, a prior study [42] employed a more interactive delivery method. In this model, parents attended concise informational sessions facilitated by trained peer educators at their children’s preschools. The sessions featured engaging visual aids, flexible scheduling, and appealing incentives for participation. This interactive approach led to high engagement rates, with an average attendance of 93%, and significantly improved parental knowledge regarding obesity-related health behaviours. These results indicate that direct engagement with parents through interactive sessions may be more effective than simply distributing materials to homes [43].

The role of communication and environmental factors in health interventions was consistently highlighted by mothers and teachers, resonating with findings from prior studies. In their 2020 phylogeographic study, Zahry et al. investigated the perspectives of low-income mothers on participating in a lifestyle intervention, identifying several facilitators that promoted engagement. These included intrapersonal factors such as access to useful information and personal motivation, interpersonal elements like communication and social connectivity, and environmental considerations, notably the availability of child-friendly intervention sites [44].

Along the same lines, a systematic review by Donglin Hu et al. (2021) examined factors influencing physical activity participation in children and adolescents using the social ecological model (SEM). Their comprehensive review of fourteen high-quality studies underscored the influence of intrapersonal factors, such as gender, age, ethnicity, and self-concept, on physical activity participation. Additionally, the review highlighted the critical role of interpersonal and organisational support from friends, parents, and teachers in fostering active engagement in physical activity [45].

Similarly, a meta-ethnographic study conducted in 2010 by Porcuna and Rodríguez-Martín analysed perceptions of physical activity among parents and teachers, identifying both facilitators and barriers. Facilitators included the recognition of physical activity benefits, active engagement, and the accommodation of children’s preferences, as well as support for active transport [46]. Conversely, barriers encompassed issues such as difficulties in quantifying physical activity, parental time constraints, cost, adverse weather conditions, traffic, long distances, and a lack of adequate and safe facilities. These insights align with the findings from our study, which suggested that the level of physical activity in children is profoundly influenced not only by individual factors but also by the broader social and school environments, as well as physical infrastructure, emphasising the need for preschool staff to consider these contextual elements in planning interventions.

Of note in our study was the significant impact of parents’ engagement and enthusiasm for the intervention programme. Parents reported that the programme had a positive effect on family relationships and overall household morale which, in turn, encouraged further participation. This observation contrasts with results from other studies which found that, despite enjoyment being derived from engaging in physical activities with their children, a segment of parents remained unaware of the importance of such involvement and assumed that fundamental motor skills would develop autonomously [47,48]. 

Participant feedback was crucial in assessing the trial procedures, particularly regarding the use of the ActiGraph GT3X accelerometer, which was deemed too invasive by many, causing discomfort among some children—a sentiment previously recorded in research involving preschool children [49]. It was found that wrist-worn devices would be preferred as they were perceived as being less intrusive while still providing reliable estimates of physical activity and sedentary behaviour. However, it is important to note that while these devices are effective in measuring overall activity levels, they do not provide postural data and their accuracy in tracking specific movements can vary [50]. Such feedback is essential for optimising device selection in interventions to balance data accuracy with participant comfort.

## 5. Strengths, Limitations, and Future Research

### 5.1. Top of Form

#### 5.1.1. Strengths

This study employed questionnaires, logbooks, and focus group discussions to assess the acceptability of the “I’m an Active Hero” (IAAH) intervention within a preschool context in Taif City, Saudi Arabia. By incorporating insights from both preschool educators and parents, the research facilitated a holistic understanding of the intervention’s reception and an evaluation of its acceptability. Adherence to the Consolidated Criteria for Reporting Qualitative Research (COREQ-32) enhanced the transparency and rigour of the study. Ethical considerations were addressed by obtaining approval from the Saudi Arabian Ministry of Health and providing a detailed methodological description, which underscored the study’s commitment to participant well-being and ethical research practices.

#### 5.1.2. Limitations

Despite its strengths, the study also has some limitations. The focus on a specific preschool setting in Taif City may constrain the generalisability of the findings to other cultural or geographical contexts, urging caution in extrapolating results. The relatively small sample size, involving two preschools and a limited number of participants in focus groups, may further impact on the exploration of diverse perspectives. An emphasis on varying recruitment strategies to encourage higher recruitment rates included the use of additional approaches, such as parental presentations, phone calls, and home visits. Potential social desirability bias in participant responses, particularly when discussing intervention acceptability, could have introduced biases and affected the accuracy of the data.

#### 5.1.3. Future Research

Several avenues for future research emerged from this study. Exploring the cultural nuances influencing intervention acceptability in Saudi Arabia would deepen our understanding, considering the specific sociocultural factors at play. Further, inclusion of a larger and more diverse participant pool across various preschools or regions would enhance generalisability and capture a broader range of perspectives. Adopting a longitudinal approach with follow-up assessments could provide insights into the sustainability of intervention acceptability, offering a comprehensive understanding of its long-term impacts. Comparative studies across different intervention models or settings, particularly RCT study designs, would also contribute to a nuanced understanding of factors influencing acceptability and effectiveness. Additionally, incorporating the perspectives of children themselves would provide valuable insights into the acceptability of the IAAH intervention from the standpoint of its primary beneficiaries. Future research avenues for this and similar studies could involve investigating the comprehensive roles of children, peers, teachers, parents, and health professionals as collaborative agents in fostering healthy energy balance-related behaviours both within the preschool environment and across broader contexts.

## 6. Conclusions

The IAAH intervention is deemed acceptable within the preschool setting based on feedback from preschool practitioners. However, further development and adaptation are required to enhance the acceptability of the home-based component. Collaborative development efforts with both parents and preschool practitioners are essential to refine the intervention’s content and delivery, and this should be prioritised before advancing to broader implementation and evaluation stages. Feasibility and acceptability studies play a crucial role in identifying challenges related to intervention acceptability. Therefore, such studies should be more extensively employed in the research and development phases of intervention evaluation.

## Figures and Tables

**Table 1:** Sociodemographic profile of participating teachers.

Characteristics		Number (n = 3)
Gender	Female	3
Degree type	Education	1
	Childhood education	2
Qualifications (highest education level)	Undergraduate	2
	Postgraduate	1
Preschool work experience	5–9 years	1
	10–14 years	2
	15 or more years	1
Role	Teachers	3
Age group	26–35	1
	36–45	1
	46–55	1

**Table 2:** Demographic characteristics of FG participating parents.

Demographic Characteristics	Total (n = 7)
Sex	
Female	3
Male	4
Marital status	
Married (men)	3
Married (women)	3
Separated (women)	1
Age group	
26–35	2
36–45	3
46–55	2
Educational level	
University or higher	6
High school or diploma	1

**Table 3:** Summary of main themes and subthemes.

Theme 1: Acceptability and Feasibility
3.3 The Need for Interventions to Increase Physical Activity in Saudi Preschools 3.3.1 Increasing opportunities
3.4. Acceptability and Feasibility 3.4.1 Practitioner perceptions of intervention content and materials 3.4.2 Parent and child perceptions 3.4.3 Training of practitioners 3.4.4 Adaptation to environmental changes in preschool intervention classrooms 3.4.5 Mode of delivery 3.4.6 Implementation of physical activity sessions by practitioners
3.5 Trial Procedures 3.5.1 Acceptance of accelerometers
3.6 Home Acceptability
3.7 Acceptability of Intervention Materials 3.7.1 Parental perceptions of materials and activities 3.7.2 Child perceptions
3.8 Trial Procedures 3.8.1 Acceptability of accelerometers
3.9 Theme 2: Outcome and Impact of the Intervention
Changes to physical activity and sedentary behaviour and enhanced focus Observation of children’s enjoyment Impact on awareness and lifestyle Perceptions of children’s leadership, confidence, and child-led activities Enhanced communication and family collaboration
3.10 Theme 3: Perceived Challenges and Potential for Sustainability
3.10.1 Barriers to delivery 3.10.2 Teachers’ prospective sustainability of IAAH 3.10.3 Parents’ prospective sustainability of IAAH
3.11 Suggestions for Improvement

**Table 4:** Acceptability scores per item for parental feedback questionnaires.

Item Number	Questionnaire Question	Percentage Coded as 1 (Agree/Strongly Agree; %)
1	Overall, did your child enjoy the activities in the programme?	94
2	Overall, did your child like the Our family’s achievements sheet provided?	65
3	Did you enjoy doing the activities with your child?	85
4	Do you think the activities helped your child be more physically active?	93
5	Do you think the activities helped your child spend less time sitting/being inactive?	86
6	Were the instructions provided for the games and activities easy to read and clear?	96

## Data Availability

The data presented in this study are available on request from the corresponding author. The data are not publicly available to maintain participant confidentiality due to the sensitive information resulting from the qualitative focus groups.

## Supplementary Materials

The following supporting information can be downloaded at: https://www.mdpi.com/article/10.3390/healthcare12141398/s1, Supplementary File S1: Consolidated Criteria for Reporting Qualitative Research (COREQ); Supplementary File S2: Teachers’ focus group guides; Supplementary File S3: Parents’ focus group guides.
