# Supplementary material for: Assessing the Acceptability of a Preschool-Based Multi-Component Physical Activity Intervention Entitled “I’m an Active Hero” (IAAH): Process Evaluation of a Feasibility Trial
Source: Healthcare (Basel). 2024 Jul 12;12(14):1398. doi: 10.3390/healthcare12141398 (PMC11275326; doi:10.3390/healthcare12141398)
Supplement: Supplementary file 1 [file healthcare-12-01398-s001.zip › Supplementary File S1 Consolidated criteria for reporting qualitative studies (COREQ).pdf]

Supplementary File S1: Consolidated criteria for reporting qualitative studies (COREQ): 32 item checklist

No. Item	Guide questions/description	Reported on Page No.
**Domain 1: Research team and reflexivity**		
Personal Characteristics		
1. Interviewer/facilitator	Which author/s conducted the interview or focus group?	Page 6
2. Credentials	What were the researcher's credentials? E.g. PhD, MD	Page 1
3. Occupation	What was their occupation at the time of the study?	Page 1
4. Gender	Was the researcher male or female?	Page 6
5. Experience and training	What experience or training did the researcher have?	Page 6
Relationship with participants		
6. Relationship established	Was a relationship established prior to study commencement?	Page 6 .
7. Participant knowledge of the interviewer	What did the participants know about the researcher? e.g. personal goals, reasons for doing the research	Page 6
8. Interviewer characteristics	What characteristics were reported about the interviewer/facilitator? e.g. Bias, assumptions, reasons and interests in the research topic	Page 1, 2, 3, 5, 6, 7, 22, 23
**Domain 2: study design**		
Theoretical framework		
9. Methodological orientation and Theory	What methodological orientation was stated to underpin the study? e.g. grounded theory, discourse analysis, ethnography, phenomenology, content analysis	Page 7
Participant selection		

10. Sampling	How were participants selected? e.g. purposive, convenience, consecutive, snowball	Page 4
11. Method of approach	How were participants approached? e.g. face-to-face, telephone, mail, email	Page 4
12. Sample size	How many participants were in the study?	Page 4 and 5
13. Non-participation	How many people refused to participate or dropped out? Reasons?	N/A
Setting		
14. Setting of data collection	Where was the data collected? e.g. home, clinic, workplace	Page 6
15. Presence of nonparticipants	Was anyone else present besides the participants and researchers?	No
16. Description of sample	What are the important characteristics of the sample? e.g. demographic data, date	Table 1
Data collection		
17. Interview guide	Were questions, prompts, guides provided by the authors? Was it pilot tested?	Page 5 and 6, Supplementary file 3
18. Repeat interviews	Were repeat interviews carried out? If yes, how many?	No
19. Audio/visual recording	Did the research use audio or visual recording to collect the data?	Page 6
20. Field notes	Were field notes made during and/or after the interview or focus group?	Page 6
21. Duration	What was the duration of the interviews or focus group?	Page 6
22. Data saturation	Was data saturation discussed?	Page 4

23. Transcripts returned	Were transcripts returned to participants for comment and/or correction?	Page 6
**Domain 3: analysis and findings**		
Data analysis		
24. Number of data coders	How many data coders coded the data?	Page 6
25. Description of the coding tree	Did authors provide a description of the coding tree?	Table 3 and 4
26. Derivation of themes	Were themes identified in advance or derived from the data?	Page 7
27. Software	What software, if applicable, was used to manage the data?	Page 6
28. Participant checking	Did participants provide feedback on the findings?	Page 7
Reporting		
29. Quotations presented	Were participant quotations presented to illustrate the themes/findings? Was each quotation identified? e.g. participant number	Table 3 and 4
30. Data and findings consistent	Was there consistency between the data presented and the findings?	Yes, there was.<br>Page 7 to 19
31. Clarity of major themes	Were major themes clearly presented in the findings?	Yes. they were.<br>From page 7 to 19
32. Clarity of minor themes	Is there a description of diverse cases or discussion of minor themes?	Discussion of major and minor themes.<br>Page 7 to 19
