# Supplementary material for: Assessing the Acceptability of a Preschool-Based Multi-Component Physical Activity Intervention Entitled “I’m an Active Hero” (IAAH): Process Evaluation of a Feasibility Trial
Source: Healthcare (Basel). 2024 Jul 12;12(14):1398. doi: 10.3390/healthcare12141398 (PMC11275326; doi:10.3390/healthcare12141398)
Supplement: Supplementary file 1 [file healthcare-12-01398-s001.zip › Supplementary File s2 Teachers focus group guides.pdf]

Supplementary file S2 : **Teacher's focus group topic guide**

The I'm an Active Hero (IAAH) Study:

Q1: To start, please tell me about your experience with the "I Am Active Hero" project and which part you generally used?

Q2: You received the Activity Guide for implementing the "I Am Active Hero" program. What are your thoughts on it, and what are your ideas about the materials included in the guide?

- How much time did you spend getting to know the materials and resources related to the "I Am Active Hero" program? Was this duration sufficient?

Q3: How did you integrate the program into the regular classroom routine? What could have made this easier for you?

Q4: Describe any challenges you encountered when delivering indoor and outdoor physical activities. [Specify the obstacles or challenges that could be faced. Ask for specific examples.]

- What solutions did you employ to address these challenges?

Q5: How effective was the training provided to you for using this program?

- What additional training or resources could have been helpful?

Q6: Regarding the "I Am Active Hero" activity records, how did you find this task (was it time-consuming, and was it user-friendly)?

Q7: The "I Am Active Hero" program includes several sessions on physical activity and passive behaviour designed to be easy to implement in the classroom or on the playground. Which ones did you specifically use and why? Was there anything you didn't use?

Q8: The "I Am Active Hero" program includes giving the child the opportunity to lead some activities. How was this aspect implemented, and what is your opinion on it?

Q9: How receptive were the children to the program's activities? What aspects of the program received a good or poor reception from the children?

Q10: What are other ways the program could have been improved?

Q11: Do you believe it's possible to continue implementing the program in the nursery in the future?

- What suggestions and ideas can support its continuity from your perspective?

Close:

Do you have any other thoughts or views you would like to share?

Thank participants for their time and inform them of what happens to the information.
