# Supplementary material for: Assessing the Acceptability of a Preschool-Based Multi-Component Physical Activity Intervention Entitled “I’m an Active Hero” (IAAH): Process Evaluation of a Feasibility Trial
Source: Healthcare (Basel). 2024 Jul 12;12(14):1398. doi: 10.3390/healthcare12141398 (PMC11275326; doi:10.3390/healthcare12141398)
Supplement: Supplementary file 1 [file healthcare-12-01398-s001.zip › Supplementary File S3 Parents focus group guides.pdf]

Supplementary file S3: **Parent/caregiver focus group topic guide.**
The I'm an Active Hero (IAAH) Study:

Q1: To begin, please tell me about your experience with the (I Am Active Hero) program and which part you generally used.

Q2: Can you describe any challenges you faced when receiving components of the (I Am Active Hero) program at home? [Specify any obstacles or challenges that may have arisen. Ask for specific examples.]

Q3: What could have made this program easier for you?

Q4: What are your thoughts on the materials you received from the nursery, such as activity cards, stickers, newsletters, and the family achievement sheet?

Q5: What suggestions do you have for improving the resources you received, including activity cards, stickers, newsletters, and the family achievement sheet?

Q6: The I Am Active Hero program includes giving the child the opportunity to lead some activities. How was this aspect implemented, and what is your opinion on it?

Q7: How receptive were the children to the program's activities? What aspects of the program received a good reception or a poor one from the children?

Q8: What other ways can the program be improved?

Q9: After implementing the I Am Active Hero program for ten weeks, did you notice any changes in your children's activity levels?

Q10: Do you expect the I Am Active Hero program to continue in the future, and what tips and suggestions do you have for its continuity from your perspective?

Close:

- Do you have any other thoughts or views you would like to share?
- What has it felt like to participate in a focus group? Is it what you expected? (If not, what did you expect?)

Thank the participants for their time and keep them updated on what happens with the information.
